# Pharmacologic de-escalation of dexamethasone during weekly paclitaxel: a randomized phase III trial evaluating safety, endocrine effects, and quality of life

**DOI:** 10.1007/s00280-026-04920-x

**Published:** 2026-07-13

**Authors:** Vanessa Armenio Scontre, Marcos Tadashi Kakitani Toyoshima, Maria Del Pilar Estevez-Diz

**Affiliations:** 1 Department of Radiology and Oncology, Instituto do Cancer do Estado de Sao Paulo – Faculdade de Medicina da Universidade de Sao Paulo, Avenida Dr. Arnaldo, 251 – 5º Andar, Sao Paulo, 01246-000 SP Brazil; 2 Endocrine Oncology Service, Instituto do Cancer do Estado de Sao Paulo – Faculdade de Medicina da Universidade de Sao Paulo, São Paulo, Brazil

**Keywords:** Breast cancer, Paclitaxel, Dexamethasone, Premedication, Steroid-sparing, Endocrine effects

## Abstract

**Objective:**

Dexamethasone is routinely used as premedication during weekly paclitaxel to prevent hypersensitivity reactions; however, prolonged corticosteroid exposure may induce clinically relevant metabolic, endocrine, and patient-reported adverse effects. This study evaluated whether selective omission of dexamethasone after the second paclitaxel infusion is safe and explored its effects on quality of life, metabolic/endocrine parameters, and short-term oncologic outcomes, including recurrence and disease-related mortality.

**Methods:**

In this prospective, randomized, open-label phase III trial, 86 women with stage I–III breast cancer receiving neoadjuvant or adjuvant AC-T or AC-TH chemotherapy were randomized 1:1 to standard dexamethasone premedication or omission after the second weekly paclitaxel infusion. The primary endpoint was safety, defined by hypersensitivity reactions and adverse events. Secondary and exploratory endpoints included patient-reported quality of life using the EORTC QLQ-C30, metabolic and endocrine parameters, and short-term oncologic outcomes. Longitudinal changes were analyzed using generalized estimating equations.

**Results:**

Eighty-four patients were included in the final analysis. No hypersensitivity reactions occurred after dexamethasone omission, and treatment completion rates were comparable between groups. After a median follow-up of 3.15 years, recurrence and disease-related mortality were similar between groups in descriptive analyses. The omission strategy was associated with improvements in role functioning and reduced worsening of pain (*p* = 0.02), constipation (*p* = 0.01), and nausea/vomiting (*p* = 0.03). No statistically significant differences were observed for emotional or physical functioning. Endocrine analyses demonstrated reduced IGF-1 levels and a trend toward lower insulin levels. These secondary analyses should be interpreted as exploratory.

**Conclusion:**

Selective omission of dexamethasone after the second weekly paclitaxel infusion was safe and well tolerated in this randomized trial. The findings support the feasibility of a corticosteroid-sparing strategy during weekly paclitaxel and suggest potential benefits in selected patient-reported and endocrine outcomes, which warrant confirmation in larger studies.

**Trial registration:**

NCT04350229, registered on 16 April 2020.

**Supplementary Information:**

The online version contains supplementary material available at 10.1007/s00280-026-04920-x.

## Introduction

Breast cancer is the most frequently diagnosed malignancy among women worldwide and remains a leading cause of cancer-related mortality [1]. In early-stage disease, curative treatment commonly includes anthracycline- and taxane-based chemotherapy, which significantly reduces the risk of recurrence and improves survival outcomes [2, 3].

Paclitaxel is a cornerstone of both adjuvant and neoadjuvant regimens and is routinely administered with corticosteroid premedication to prevent hypersensitivity reactions and mitigate chemotherapy-related symptoms [4]. Traditionally, dexamethasone has been maintained throughout the entire course of weekly paclitaxel.

However, accumulating evidence suggests that hypersensitivity reactions to paclitaxel occur predominantly during the initial infusions, raising questions about the necessity of prolonged corticosteroid exposure [5–7]. Recent randomized data have further supported this concept, demonstrating that lower doses of dexamethasone are non-inferior to standard dosing for the prevention of paclitaxel-related hypersensitivity reactions, reinforcing that prolonged or high-dose corticosteroid exposure may be unnecessary [8]. Extended dexamethasone use is associated with well-recognized adverse effects, including insomnia, mood changes, fluid retention, gastrointestinal discomfort, and musculoskeletal symptoms, which may negatively influence treatment tolerance and patient-reported quality of life [9]. These effects may be particularly relevant in extended weekly taxane schedules, where cumulative corticosteroid exposure can be substantial.

Beyond their clinical effects, corticosteroids exert broad metabolic and endocrine modulation, including alterations in glucose metabolism, insulin signaling, and hypothalamic–pituitary–adrenal axis regulation. These pharmacologic effects may influence treatment tolerance, systemic inflammation, and overall physiological resilience during chemotherapy. Therefore, minimizing unnecessary corticosteroid exposure represents not only a supportive care strategy but also a pharmacologically relevant intervention with potential systemic implications.

In parallel with these developments, observational studies and simplified premedication protocols have explored broader premedication de-escalation strategies after the initial paclitaxel infusions in patients who do not experience hypersensitivity reactions [5–7, 10, 11]. These studies suggest that routine continuation of full premedication throughout treatment may not be necessary in selected patients. In addition, the ongoing DEXASTOP trial was designed to prospectively evaluate omission of dexamethasone in this setting and may provide further evidence regarding corticosteroid de-escalation strategies [12].

However, most available data evaluate premedication omission as a combined strategy, making it difficult to isolate the specific pharmacological and systemic effects attributable to corticosteroid exposure. Given the well-established metabolic, endocrine, and patient-reported effects of glucocorticoids, understanding the independent contribution of dexamethasone remains clinically relevant.

Therefore, prospective randomized data specifically addressing dexamethasone omission—particularly with detailed evaluation of longitudinal patient-reported outcomes and metabolic parameters—remain limited.

In this randomized phase III trial, we evaluated whether omission of dexamethasone after the second weekly paclitaxel infusion is safe and whether it influences quality of life, symptom burden, and endocrine parameters over time in patients with stage I–III breast cancer receiving AC-T or AC-TH chemotherapy.

These findings provide pharmacologic rationale for steroid-sparing strategies and may contribute to reducing unnecessary corticosteroid exposure during weekly taxane therapy.

## Methods

### Study design and ethical approval

This prospective, randomized, open-label phase III trial was conducted at a single tertiary cancer center (Instituto do Câncer do Estado de São Paulo, ICESP). Eligible participants were women aged ≥ 18 years with stage I–III invasive breast cancer, Eastern Cooperative Oncology Group (ECOG) performance status 0–1, corresponding approximately to a Karnofsky Performance Status ≥ 80%, and adequate renal, hepatic, and cardiac function. Participants were randomized in a 1:1 ratio using the REDCap electronic data capture system. Randomization was performed using permuted blocks with variable block sizes through the REDCap system to ensure allocation concealment.

Baseline clinical characteristics such as age, tumor stage, hormone receptor status, histologic grade, comorbidities, and treatment setting were recorded to describe the study population and assess balance between groups after randomization. Variables such as treatment setting and type of surgery were included to provide a comprehensive clinical characterization of the study population and to ensure transparency in the reporting of baseline characteristics.

Patients received neoadjuvant or adjuvant chemotherapy with anthracycline- and taxane-based regimens, including AC-T or AC-TH, according to institutional protocols [13, 14]. Exclusion criteria included metastatic disease, diabetes mellitus, chronic use of corticosteroids or non-steroidal anti-inflammatory drugs, and cognitive impairment. The use of other immunomodulatory agents, such as conventional immunosuppressants or biological therapies, was not systematically recorded. Chronic antihistamine use was also not prospectively captured.

The study was approved by the institutional ethics committee (CAAE **31965920.0.0000.0065**) and registered at ClinicalTrials.gov (NCT04350229).

### Treatment protocols

Patients received one of the following chemotherapy regimens:

- AC-T: doxorubicin 60 mg/m² plus cyclophosphamide 600 mg/m² every 21 days for four cycles, followed by weekly paclitaxel 100 mg/m² for eight cycles.
- AC-TH: doxorubicin 60 mg/m² plus cyclophosphamide 600 mg/m² every 21 days for four cycles, followed by weekly paclitaxel 80 mg/m² for 12 cycles combined with trastuzumab (6 mg/kg every 21 days for 17 cycles).

All patients received standard premedication for paclitaxel during the first two infusions, including dexamethasone, antihistamines, and H2-receptor antagonists.

Dexamethasone was administered intravenously at a dose of 10 mg approximately 30 min prior to paclitaxel infusion during the first two cycles. Antihistamines and H2-receptor antagonists were administered according to institutional protocols. No additional corticosteroids were routinely administered before or after infusion unless clinically indicated.

Paclitaxel was administered as a 1-hour intravenous infusion, diluted in 250 mL of normal saline or 5% dextrose, according to institutional protocols. No routine oral corticosteroids were administered on the days preceding or following paclitaxel infusion unless clinically indicated.

Patients who experienced hypersensitivity reactions during the first two paclitaxel infusions were managed according to institutional protocols and remained eligible for inclusion, provided that continuation of paclitaxel was clinically appropriate.

Participants were randomized 1:1 using REDCap to either:

- Control group: standard dexamethasone premedication before every weekly paclitaxel infusion; or.
- Experimental group: omission of dexamethasone after the second weekly paclitaxel infusion.

**Fig. 1:**
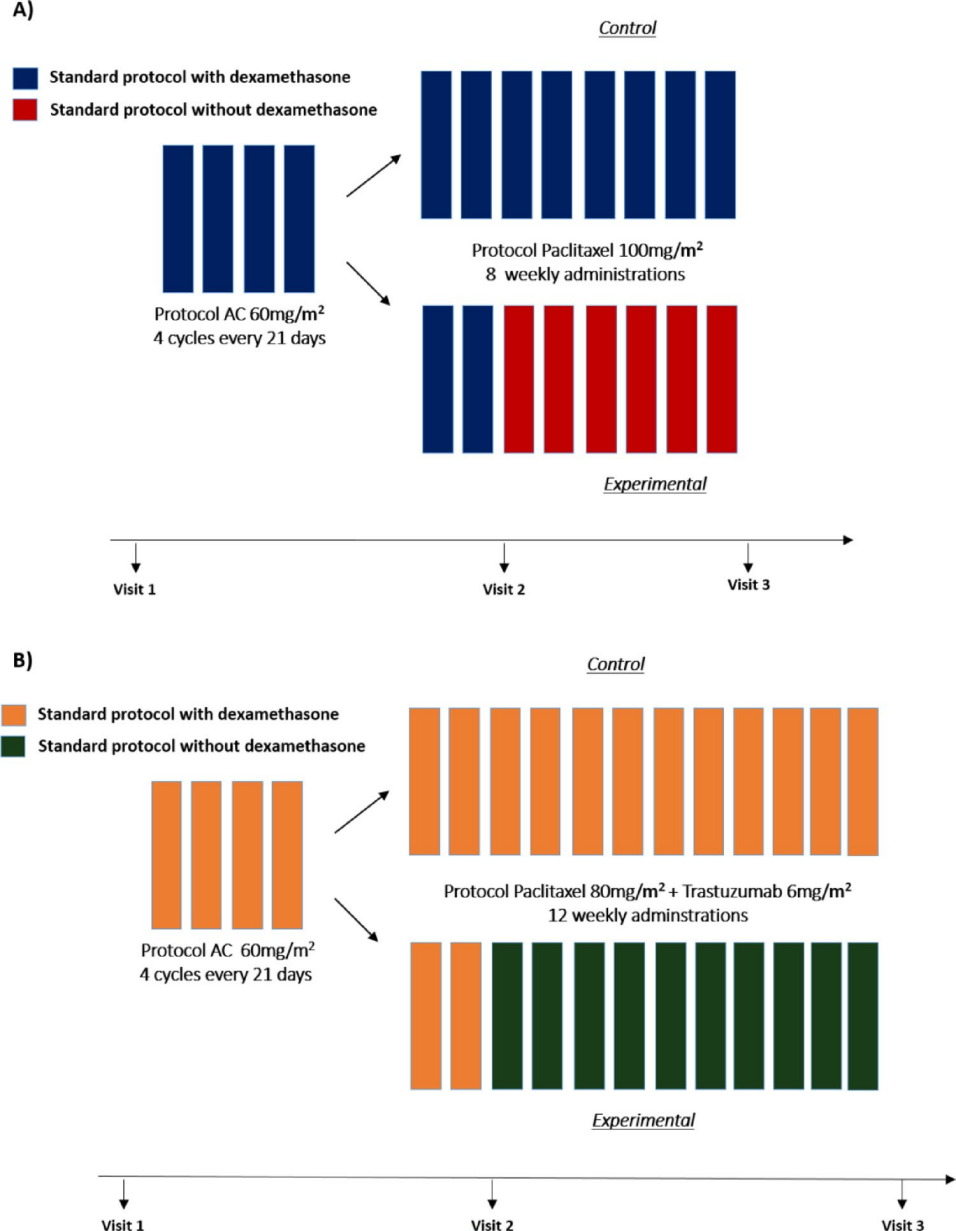
Study design, treatment allocation, and timing of assessments. Patients were randomized 1:1 to standard dexamethasone premedication before every weekly paclitaxel infusion or to dexamethasone omission after the second weekly paclitaxel infusion. Study assessments were performed at three predefined time points: Visit 1 (baseline, before chemotherapy), Visit 2 (before the third weekly paclitaxel infusion), and Visit 3 (24 h after the final paclitaxel infusion). Hypersensitivity reactions and adverse events were monitored throughout all paclitaxel infusions and during the treatment period. QoL was assessed using the EORTC QLQ-C30 at Visits 1, 2, and 3

### Assessments and follow-up

Clinical evaluations, laboratory tests, and patient-reported outcomes were performed at three predefined study time points: Visit 1 (baseline, before initiation of systemic chemotherapy), Visit 2 (immediately before the third weekly paclitaxel infusion), and Visit 3 (24 h after the final paclitaxel infusion).

Hypersensitivity reactions were monitored prospectively during every paclitaxel infusion and recorded throughout the treatment period, rather than only at a single post-infusion time point. All infusion-related reactions and other adverse events were graded according to the Common Terminology Criteria for Adverse Events (CTCAE), version 5.0. Corticosteroid-related adverse effects were defined a priori as adverse events commonly associated with systemic glucocorticoid exposure, including insomnia, mood changes, gastrointestinal discomfort, fluid retention, hyperglycemia, and musculoskeletal symptoms. These events were prospectively recorded during clinical assessments throughout treatment and graded according to CTCAE version 5.0 when applicable.

In addition, symptom burden potentially related to corticosteroid exposure was evaluated through patient-reported outcomes using the EORTC QLQ-C30 symptom scales.

Quality of life was assessed using the EORTC QLQ-C30 questionnaire at Visit 1, Visit 2, and Visit 3, allowing longitudinal comparison of patient-reported outcomes from baseline to the completion of paclitaxel treatment.

A schematic representation of the study design, treatment schedule, and timing of assessments is provided in Fig. 1.

### Outcomes

The primary endpoint of the study was safety, defined as the occurrence of paclitaxel-related hypersensitivity reactions and treatment-related adverse events recorded throughout treatment. A key secondary endpoint was patient-reported quality of life (QoL), assessed longitudinally using the EORTC QLQ-C30 questionnaire at baseline, before the third weekly paclitaxel infusion, and 24 h after the final paclitaxel infusion.

Secondary endpoints included treatment tolerability, laboratory and metabolic parameters, body composition assessed by bioimpedance, and short-term oncologic outcomes such as recurrence and mortality. Given the single-center design and limited sample size, these oncologic outcomes were evaluated descriptively and should be considered exploratory rather than confirmatory.

### Statistical analysis

The study was primarily designed to evaluate safety outcomes. Sample size calculation was based on an expected reduction in corticosteroid-related adverse effects following dexamethasone omission.

Based on previous observational studies evaluating dexamethasone omission during weekly paclitaxel [5, 7], a reduction from 50% to 20% was assumed following dexamethasone omission. A total of 76 patients were required to achieve 80% power with a two-sided α of 0.05. To account for potential attrition, 86 patients were enrolled.

Quality-of-life outcomes were analyzed longitudinally using generalized estimating equations (GEE). Generalized estimating equation (GEE) models were fitted assuming an exchangeable correlation structure, with a Gaussian distribution and identity link function for continuous outcomes. Models included time, treatment group, and the interaction between time and treatment group. Quality-of-life analyses were conducted using available-case data. Questionnaire completion rates at each time point were high, and no imputation procedures were performed. Because the study was primarily powered for safety outcomes, quality-of-life analyses were pre-specified but not separately powered and should therefore be interpreted as exploratory. Continuous variables were compared using Student’s t test or the Mann–Whitney U test, as appropriate, and categorical variables were analyzed using chi-square or Fisher’s exact tests. Additional delta analyses comparing Visit 3 and Visit 2 scores were performed for QoL outcomes. No formal multiplicity adjustment was performed for secondary or exploratory endpoints; therefore, these analyses should be interpreted cautiously. Statistical significance was defined as *p* < 0.05.

All analyses were performed using Stata version 15 (StataCorp, College Station, TX, USA). The statistical analysis plan was predefined prior to data analysis.

Because the study was not designed as a non-inferiority trial for oncologic outcomes and the number of recurrence events was limited, formal time-to-event analyses (e.g., Kaplan–Meier curves or hazard ratios) were not performed. Recurrence and mortality were therefore summarized descriptively.

Given the study design and sample size, the trial was not powered for formal pharmacokinetic or pharmacodynamic analyses; however, exploratory endocrine and metabolic markers were included to provide biological context for corticosteroid exposure.

## Results

### Patient population

Between May 2020 and June 2021, 86 women with stage I–III breast cancer were enrolled and randomized in a 1:1 ratio to the control group (standard dexamethasone premedication) or the experimental group (dexamethasone omission after the second paclitaxel infusion). Two patients in the experimental group were excluded due to COVID-19 infection requiring systemic corticosteroid therapy. Consequently, 84 patients were included in the final analysis (43 control and 41 experimental).

**Fig. 2:**
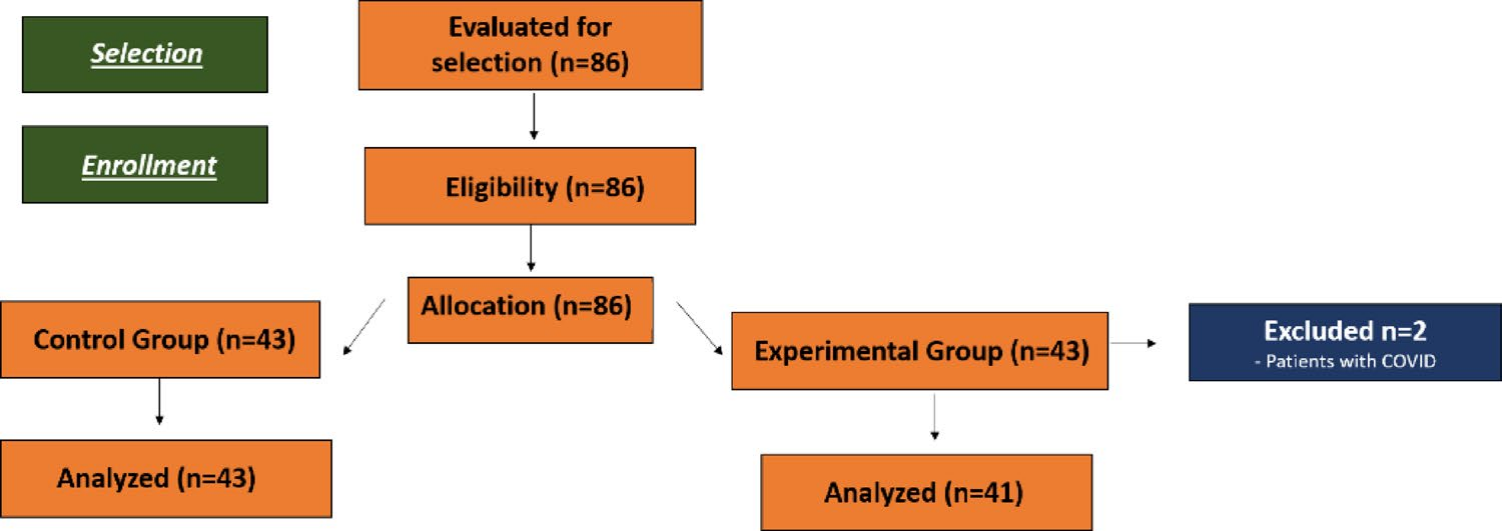
CONSORT flow diagram of patient enrollment, randomization, and analysis

The CONSORT flow diagram is shown in Fig. 2. Baseline characteristics were well balanced between groups (Table 1). Baseline quality-of-life scores assessed using the EORTC QLQ-C30 were comparable between groups across all domains, as detailed in **Supplementary Table S1**. Most patients had stage II disease, invasive ductal carcinoma, intermediate histologic grade, and a luminal molecular subtype. The distribution of comorbidities, menopausal status, treatment setting (neoadjuvant vs. adjuvant), and surgical approach did not differ significantly between groups.

**Table 1:** Baseline demographic and clinical characteristics of the study population

	Control	Experimental
	(*n* = 43)	(*n* = 41)
**Age (years)**	47.0 ± 12.1	48.1 ± 11
**Weight (kg)**	78.9 ± 19.7	79.6 ± 19.7
**Race**		
White	28 (65.1%)	25 (60.9%)
Hispanic	4 (9.3%)	9 (21.9%)
Black	9 (20.9%)	7 (17.0%)
Asian	2 (4.7%)	0
**Comorbidities**		
None	29 (67.4%)	25 (60.9%)
Arterial Hypertension	13 (30.2%)	12 (29.2%)
Psychiatric Disorder	1 (2.3%)	2 (4.8%)
Smoking History	0	2 (4.8%)
**Hormone Status**		
Pre Menopause	26 (60.5%)	25 (60.9%)
Post Menopause	17 (39.5%)	16 (39.0%)
**Staging**		
IA	0	1 (2.4%)
IB	0	0
IIA	7 (16.3%)	6 (14.6%)
IIB	19 (44.2%)	18 (43.9%)
IIIA	9 (20.9%)	9 (21.9%)
IIIB	7 (16.3%)	5 (12.1%)
IIIC	1 (2.3%)	2 (4.8%)
**Histological Type**		
Ductal Carcinoma	40 (93.0%)	39 (95.1%)
Lobular Carcinoma	3 (7.0%)	2 (4.8%)
**Histological Grade**		
G1	1 (2.3%)	0
G2	30 (69.8%)	28 (68.2%)
G3	12 (27.9%)	13 (31.7%)
**Immunohistochemistry**		
Luminal A	4 (9.3%)	7 (17.0%)
Luminal B	31 (72.0%)	25 (60.9%)
Triple negative	6 (14%)	7 (17.0%)
Her2 Enriched	2 (4.7%)	2 (4.8%)
**Treatment**		
Neoadjuvant	26 (60.5%)	19 (46.3%)
Adjuvant	17 (39.5%)	22 (53.6%)
**Type of Surgery**		
Quadrantectomy	28 (65.1%)	24 (58.5%)
Mastectomy	15 (34.9%)	17 (41.4%)

Values are presented as mean ± standard deviation, median (interquartile range), or number (percentage), as appropriate

Molecular subtypes were defined as follows: Luminal A (HR-positive, HER2-negative, low proliferation index), Luminal B (HR-positive with high proliferation index and/or HER2-positive), HER2-enriched (HR-negative, HER2-positive), and triple-negative (HR-negative, HER2-negative)

Breast cancer staging was based on the AJCC TNM classification, 8th edition (2017). Baseline characteristics are presented descriptively to demonstrate the comparability of the treatment groups after randomization. Consistent with CONSORT recommendations for randomized clinical trials, no formal statistical comparisons were performed for baseline variables

### Oncologic outcomes and safety

After a median follow-up of 3.15 years (interquartile range [IQR], 2.92–3.38), disease recurrence occurred in eight patients (19%) in the control group and nine patients (22%) in the experimental group. Mortality related to disease progression occurred in four patients (9%) in the control group and four patients (10%) in the experimental group. These findings were analyzed descriptively given the limited number of events. No hypersensitivity reactions were observed during paclitaxel administration in either group. No adverse events attributable to dexamethasone omission were observed. Treatment completion rates were comparable between groups.

### Laboratory parameters

Changes in laboratory parameters between Visit 2 (before the third paclitaxel infusion) and Visit 3 (24 h after the final infusion) are summarized in Table 2. Given the exploratory nature of these analyses and the relatively small sample size, these findings should be interpreted cautiously and considered hypothesis-generating.

**Table 2:** Changes in laboratory parameters between Visit 2 and Visit 3

Variable	Control			Experimental		
	Visit 2	Visit 3	Variation (delta)	Visit 2	Visit 3	Variation (delta)
Leukocytes (×10⁹/L)	4.15 ± 2.59	4.48 ± 1.28	0.33	3.84 ± 1.48	4.28 ± 1.48	0.44
Neutrophils (×10⁹/L)	2.41 ± 1.00	2.86 ± 1.03	0.45	2.42 ± 1.14	2.64 ± 1.10	0.22
Platelets (×10⁹/L)	270 ± 73	287 ± 81	17	283 ± 66	324 ± 55	41
Neutrophil/Leukocyte Ratio	0.62 ± 0.11	0.62 ± 0.10	0.04	0.61 ± 0.09	0.60 ± 0.09	-0.01
Aldosterone (ng/dL)	11.20 ± 9.79	11.51 ± 11.10	0.31	10.97 ± 7.02	10.01 ± 5.70	-0.96
Indirect Bilirubin (mg/dL)	0.22 ± 0.15	0.20 ± 0.14	-0.02	0.18 ± 0.11	0.1 ± 0.09	-0.08
Direct Bilirubin (mg/dL)	0.15 ± 0.07	0.15 ± 0.06	0.00	0.13 ± 0.05	0.14 ± 0.04	0.00
Growth Hormone (ng/mL)	0.51 ± 0.65	0.55 ± 0.83	-0.04	0.54 ± 0.83	0.54 ± 0.78	0.00
IGF-1 (ng/mL)	151.85 ± 59.01	162.29 ± 70.69	10.44	158.59 ± 61.90	128.22 ± 53.39	-30.37
Insulin (µIU/mL)	24.52 ± 17.36	24.92 ± 20.41	0.40	23.37 ± 15.97	18.97 ± 12.09	-4.40
Potassium (mEq/L)	4.21 ± 0.36	4.12 ± 0.40	-0.09	4.24 ± 0.36	4.16 ± 0.32	-0.08
LDH (U/L)	214.12 ± 57.98	217.48 ± 51.70	3.36	194.88 ± 37.93	219.61 ± 41.88	24.73
Total Protein (g/dL)	6.83 ± 0.45	8.22 ± 0.93	1.39	6.83 ± 0.88	7.00 ± 0.53	0.17
ACTH (pg/mL)	19.13 ± 11.17	20.84 ± 10.29	1.71	22.40 ± 10.60	28.06 ± 17.91	5.66
C-Reactive Protein (mg/L)	7.83 ± 8.04	8.25 ± 9.87	0.42	6.09 ± 5.10	6.35 ± 4.92	0.26

Values are presented as mean ± standard deviation or median (interquartile range), as appropriate. Changes (delta values) represent Visit 3 minus Visit 2, where Visit 2 was performed before the third weekly paclitaxel infusion and Visit 3 was performed 24 h after the final infusion. Results should be interpreted with caution due to variability, the relatively small sample size, and the exploratory nature of these analyses

IGF-1, insulin-like growth factor 1; ACTH, adrenocorticotropic hormone; LDH, lactate dehydrogenase

In the experimental group, insulin-like growth factor 1 (IGF-1) levels decreased, whereas insulin levels showed a downward trend. Adrenocorticotropic hormone ACTH levels increased modestly in the experimental group, whereas lower values were observed in the control group. Although this pattern is biologically consistent with corticosteroid-related hypothalamic–pituitary–adrenal axis suppression, these findings were exploratory and should be interpreted cautiously.

Platelet counts increased in both groups over the study period, with a greater increase observed in the experimental group. Lactate dehydrogenase (LDH) levels showed an upward trend in the experimental group. Other laboratory parameters, including leukocyte count, neutrophils, bilirubin, aldosterone, potassium, total protein, and C-reactive protein, demonstrated no clinically meaningful differences between groups.

### Bioimpedance analysis

Bioimpedance results are presented in Table 3. Body weight, body mass index, body surface area, and fat mass remained stable in both groups throughout the study period. These analyses were exploratory and not powered for formal hypothesis testing.

**Table 3:** Changes in body composition assessed by bioimpedance analysis between Visit 2 and Visit 3

Variable	Control			Experimental		
	Visit 2	Visit 3	Variation (delta)	Visit 2	Visit 3	Variation (delta)
Weight (kg)	78.24 ± 19.78	81.34 ± 19.78	3.10	76.11 ± 16.17	78.73 ± 15.88	2.62
Body surface area (m^2^)	1.80 ± 0.23	1.81 ± 0.23	0.00	1.75 ± 0.18	1.75 ± 0.18	0.00
Body mass index (kg/m²)	30.93 ± 6.57	31.17 ± 6.57	0.24	29.60 ± 5.85	29.41 ± 5.71	-0.19
Lean mass (kg)	45.01 ± 8.29	45.38 ± 8.79	0.37	43.60 ± 6.28	44.46 ± 6.07	0.86
Lean mass percentage (%)	58.60 ± 6.77	58.92 ± 6.72	0.32	59.37 ± 6.35	60.98 ± 6.44	1.61
Fat mass (kg)	33.85 ± 12.99	33.91 ± 13.00	-0.04	30.45 ± 10.99	30.45 ± 11.01	-0.15
Fat mass percentage (%)	41.50 ± 7.04	41.48 ± 6.72	0.09	40.07 ± 6.34	39.02 ± 6.44	-0.99

Values are presented as mean ± standard deviation or median (interquartile range), as appropriate. Changes represent differences between Visit 2 (before the third weekly paclitaxel infusion) and Visit 3 (24 h after the final infusion). Between-group comparisons were performed using generalized estimating equations

**Fig. 3:**
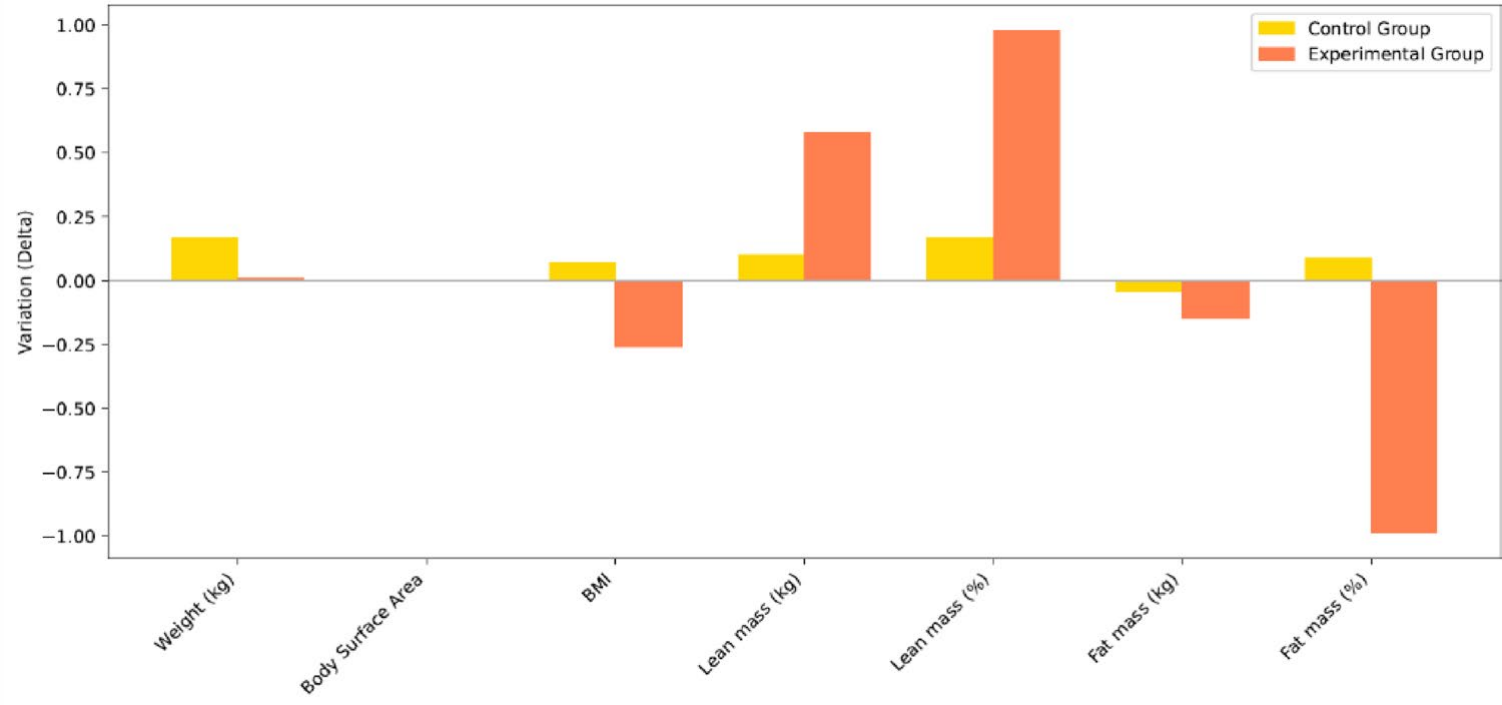
Changes in body composition assessed by bioimpedance analysis. Longitudinal changes in body composition parameters between Visit 2 (before the third paclitaxel infusion) and Visit 3 (24 h after the final infusion) in the control and experimental groups. Data include body weight, body mass index (BMI), lean mass, and fat mass. Values are presented as mean changes (delta)

A non-significant trend toward preservation and slight increase in lean mass was observed in the experimental group, whereas lean mass remained stable in the control group. Changes in body composition over time are illustrated in Fig. 3.

### Quality of life (EORTC QLQ-C30)

**Fig. 4:**
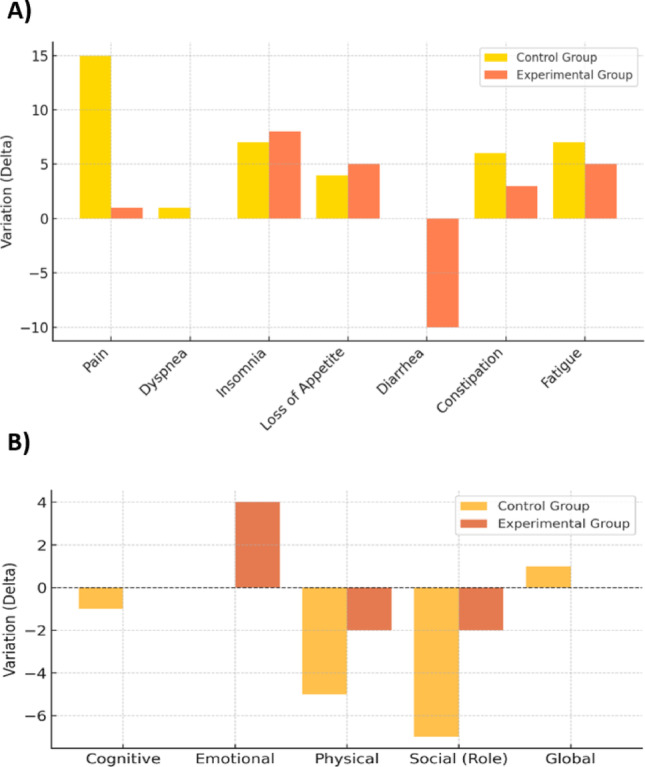
Longitudinal changes in quality-of-life domains assessed by the EORTC QLQ-C30 questionnaire. (**A**) Functional scale variations, including emotional, physical, and social (role) functioning, expressed as changes between Visit 2 and Visit 3. (**B**) Symptom scale variations, including pain, nausea and vomiting, constipation, fatigue, insomnia, diarrhea, and dyspnea. Higher scores on functional scales indicate better functioning, whereas higher scores on symptom scales indicate greater symptom burden. Values are presented as mean changes (delta)

Quality-of-life outcomes assessed using the EORTC QLQ-C30 questionnaire demonstrated selective differences favoring dexamethasone omission (Table 4). Between Visits 2 and 3, a statistically significant between-group difference was observed for role functioning (*p* = 0.03). Functional scale trajectories are depicted in Fig. 4.

**Table 4:** Changes in quality-of-life scores assessed by the EORTC QLQ-C30 questionnaire between Visit 2 and Visit 3

EORTC QLQ-C30	Control Group			Experimental Group			*p*-value
	Visit 2	Visit 3	Variation (delta)	Visit 2	Visit 3	Variation (delta)	
Cognitive	68.60 (28.69)	66.67 (28.64)	-1.94 (26.28)	65.04 (31.80)	69.51 (28.84)	4.47 (24.45)	0.09
Emotional	54.84 (33.34)	54.84 (29.62)	0 (26.16)	69.11 (27.59)	67.28 (31.86)	-1.83 (23.97)	0.62
Physical	73.33 (16.84)	67.91 (17.77)	-5.43 (17.11)	72.36 (19.30)	73.66 (20.81)	1.30 (20.12)	0.07
Role	70.54 (25.93)	56.59 (30.02)	-13.95 (25.18)	65.85 (30.95)	66.67 (27.39)	0.81 (28.37)	0.03
Nausea and vomiting	21.71 (27.35)	20.93 (27.24)	-0.78 (22.70)	18.29 (24.38)	8.13 (14.49)	-10.16 (22.63)	0.03
Pain	28.68 (31.14)	34.11 (26.96)	5.43 (25.39)	33.74 (30.16)	30.08 (29.16)	-3.66 (26.75)	0.02
Dyspnea	17.83 (22.24)	24.03 (26.55)	6.20 (25.46)	11.38 (16.00)	19.51 (30.71)	8.13 (25.58)	0.80
Insomnia	41.86 (35.70)	44.96 (36.28)	3.10 (36.96)	28.46 (29.40)	31.71 (35.71)	3.25 (32.32)	0.86
Appetite Loss	20.16 (29.22)	27.91 (31.65)	7.75 (39.06)	17.89 (26.97)	24.39 (29.84)	6.50 (37.43)	0.72
Diarrhea	11.63 (24.00)	15.50 (29.41)	3.88 (27.42)	9.76 (23.86)	12.20 (26.62)	2.44 (31.08)	0.84
Constipation	25.58 (32.40)	31.78 (36.34)	6.20 (27.46)	35.77 (40.41)	23.58 (34.35)	-12.20 (39.97)	0.01
Fatigue	34.88 (28.13)	42.12 (25.61)	7.24 (31.60)	33.33 (35.75)	35.50 (27.80)	2.17 (47.48)	0.77
Overall score	25.39 (18.36)	25.97 (18.92)	0.58 (19.53)	33.13 (17.33)	32.93 (18.06)	-0.20 (16.61)	0.60

Values are presented as mean ± standard deviation or median (interquartile range), as appropriate. Changes represent differences between Visit 2 (before the third weekly paclitaxel infusion) and Visit 3 (24 h after the final infusion). Between-group comparisons were performed using generalized estimating equations

Higher scores on functional scales indicate better functioning, whereas higher scores on symptom scales indicate greater symptom burden

Symptom scales also favored the experimental group, with significantly less worsening of nausea and vomiting (*p* = 0.03), pain (*p* = 0.02), and constipation (*p* = 0.01). No statistically significant differences were observed for emotional functioning (*p* = 0.62), physical functioning (*p* = 0.07), fatigue, insomnia, appetite loss, diarrhea, or dyspnea. Symptom trajectories are shown in Fig. 4.

## Discussion

This randomized phase III trial demonstrates that selective omission of dexamethasone after the second weekly paclitaxel infusion is safe and well tolerated. No evidence of compromised short-term oncologic outcomes was observed; however, these findings should be interpreted as exploratory given the limited number of events, consistent with prior studies evaluating paclitaxel safety and premedication strategies [5–7]. Hypersensitivity reactions to paclitaxel are known to occur predominantly during the first infusions, largely related to the solvent Cremophor EL rather than to cumulative exposure [15, 16]. In line with previous observational studies and simplified premedication protocols [10, 11], no hypersensitivity reactions were observed after dexamethasone withdrawal in the present study, reinforcing the biological plausibility and clinical safety of a steroid-sparing approach during weekly paclitaxel therapy. In addition to maintaining safety, dexamethasone omission was associated with favorable patient-reported outcomes, which represent one of the principal findings of the present study.

Quality-of-life outcomes represent one of the most important contributions of this study. The magnitude of the observed changes was interpreted in the context of established minimal important differences for the EORTC QLQ-C30, commonly around 10 points depending on the domain. In this context, the between-group difference in change for role functioning exceeded this threshold, suggesting a clinically meaningful benefit favoring dexamethasone omission. Constipation also showed a difference exceeding the MID threshold, supporting potential clinical relevance. By contrast, the between-group differences observed for nausea/vomiting and pain were close to, but slightly below, the commonly used 10-point threshold; therefore, these findings should be interpreted as suggestive rather than clearly clinically meaningful. While dexamethasone is traditionally used to mitigate chemotherapy-related symptoms, prolonged exposure has been associated with neuropsychiatric effects, gastrointestinal discomfort, and altered pain perception [9, 17]. Experimental and clinical data suggest that glucocorticoids may shift from a protective to a deleterious gastrointestinal profile with prolonged use, particularly in vulnerable populations [18, 19]. The relatively low Global Health Status scores should be interpreted in the context of active chemotherapy, as assessments were performed during paclitaxel treatment rather than at baseline or after treatment completion.

Laboratory analyses revealed differences in metabolic and endocrine parameters consistent with reduced corticosteroid exposure. The experimental group showed a reduction in IGF-1 levels and a downward trend in insulin concentrations. These findings were exploratory and should be considered hypothesis-generating [20]. In contrast, lower ACTH levels were observed in the control group. Although this pattern is biologically consistent with hypothalamic–pituitary–adrenal axis suppression associated with prolonged dexamethasone exposure [21], the observed between-group difference was exploratory and should be interpreted cautiously.

Although these findings were exploratory and not powered for definitive metabolic conclusions, they support the biological rationale for minimizing unnecessary corticosteroid exposure during chemotherapy.

These findings reinforce the pharmacological relevance of corticosteroid exposure during chemotherapy. The observed reduction in IGF-1 and trend toward lower insulin levels in the omission group suggest attenuation of glucocorticoid-induced metabolic dysregulation, a well-described consequence of systemic corticosteroid use. Although exploratory, these results provide biological support for steroid de-escalation strategies and highlight the systemic impact of premedication regimens beyond hypersensitivity prevention. Some laboratory parameters showed substantial variability, likely reflecting outliers in a relatively small sample size.

Hematologic parameters also differed modestly between groups. Platelet counts increased in both arms, with a greater rise observed in the experimental group. Corticosteroids are known to modulate hematopoiesis and inflammatory signaling through complex and sometimes paradoxical mechanisms [22]. The clinical significance of this finding remains uncertain, particularly in the absence of thrombotic events or differences in oncologic outcomes. Similarly, the non-significant increase in lactate dehydrogenase observed in the experimental group likely reflects nonspecific metabolic or inflammatory variation rather than clinically relevant tissue injury [23].

Body composition analyses showed overall stability in weight, body mass index, and fat mass in both groups. A trend toward preservation of lean mass was observed in the experimental group, although it did not reach statistical significance. Given the well-established catabolic effects of prolonged corticosteroid exposure on skeletal muscle, including inhibition of protein synthesis and increased proteolysis [24], this finding is clinically relevant and warrants further investigation in larger cohorts.

Several limitations should be acknowledged. This was a single-center, open-label study with a relatively modest sample size, which may limit the generalizability of the findings. The lack of blinding represents an important limitation, particularly for patient-reported outcomes. Because participants were aware of whether dexamethasone had been omitted, expectations regarding symptom burden, treatment tolerability, or quality of life may have influenced questionnaire responses. This awareness could have introduced a bias favoring the experimental group, potentially leading to an overestimation of the magnitude of the observed quality-of-life benefits. However, the consistency of the findings across multiple symptom domains and the absence of differences in objective safety outcomes provide some reassurance regarding the overall validity of the results.

In addition, the use of other immunomodulatory agents and chronic antihistamine therapy was not prospectively recorded. Although such treatments were not routinely expected in this population, their potential influence on hypersensitivity reactions or patient-reported outcomes cannot be completely excluded.

The absence of formal pharmacokinetic and pharmacodynamic assessments represents another study constraint and should be addressed in future investigations to better characterize the exposure–response relationship of corticosteroid modulation during taxane therapy.

Despite these limitations, this trial provides prospective randomized evidence supporting a steroid-sparing strategy during weekly paclitaxel therapy. The combination of maintained oncologic safety and improved quality of life suggests that routine, prolonged dexamethasone premedication may not be necessary for all patients. Future multicenter studies with larger sample sizes and extended follow-up are needed to confirm these findings and to refine individualized supportive care strategies in breast cancer chemotherapy.

## Conclusion

Selective omission of dexamethasone after the second weekly paclitaxel infusion was safe and well tolerated in patients with stage I–III breast cancer receiving AC-T or AC-TH chemotherapy. No hypersensitivity reactions or safety concerns were observed, and short-term oncologic outcomes, including recurrence and mortality, were comparable between the dexamethasone-omission and standard premedication groups.

Importantly, dexamethasone omission was associated with improvements in role functioning and reduced worsening of pain, constipation, and nausea and vomiting. Favorable trends in metabolic and endocrine parameters further support the biological plausibility of minimizing prolonged corticosteroid exposure during weekly taxane therapy.

These findings suggest that routine continuation of dexamethasone throughout all paclitaxel infusions may not be necessary for all patients and that a more selective, individualized premedication strategy could improve treatment tolerability without compromising safety. Larger multicenter studies with longer follow-up are warranted to confirm these results.

## Electronic Supplementary Material

Below is the link to the electronic supplementary material.

Supplementary Material 1

## Data Availability

No datasets were generated or analysed during the current study.
