# Supplementary material for: Pharmacologic de-escalation of dexamethasone during weekly paclitaxel: a randomized phase III trial evaluating safety, endocrine effects, and quality of life
Source: Cancer Chemother Pharmacol. 2026 Jul 13;96(1):75. doi: 10.1007/s00280-026-04920-x (PMC13364796; doi:10.1007/s00280-026-04920-x)
Supplement: Supplementary file 1 — Supplementary Material 1 [file 280_2026_4920_MOESM1_ESM.docx]

**Supplementary Table S1**. Baseline Quality-of-Life Scores (EORTC QLQ-C30)

Experimental

Control

| **EORTC QLQ-C30** |  |  |
| --- | --- | --- |
|  | **Visit 1** | **Visit 1** |
| Cognitive | 71.54 | 77.64 |
| Emotional | 63.21 | 60.77 |
| Physical | 82.76 | 81.95 |
| Role | 69.51 | 76.42 |
| Nausea and vomiting | 7.72 | 8.53 |
| Pain | 28.04 | 26.82 |
| Dyspnea | 11.38 | 8.13 |
| Insomnia | 27.52 | 25.19 |
| Appetite Loss | 11.38 | 7.31 |
| Diarrhea | 8.53 | 9.30 |
| Constipation | 21.95 | 15.44 |
| Fatigue | 20.05 | 24.39 |
| Overall score | 26.01 | 28.04 |

Values are presented as mean scores. Baseline comparisons are descriptive and no formal statistical testing was performed. Higher scores on functional scales indicate better functioning, whereas higher scores on symptom scales indicate greater symptom burden.
